# Cannabis Use Disorder Is Associated With Increased Risk of Acute Myocardial Infarction in Adults With Metabolic Dysfunction-Associated Steatohepatitis (MASH) Cirrhosis: A Population-Based Analysis

**DOI:** 10.7759/cureus.103299

**Published:** 2026-02-09

**Authors:** Basile Njei, Sarpong Boateng, Ifeoma Kwentoh, Prince Ameyaw, Chukwunonso Ezeani, Nso Nso, Sabastian Forsah, Christian A Dimala, Derek Fan Ugwendum, Lea-Pearl Njei, Yazan A Al-Ajlouni, Joseph K Lim, Jonathan A Dranoff

**Affiliations:** 1 Medicine, Yale University School of Medicine, New Haven, USA; 2 Engelhardt School of Global Health and Bioethics, Euclid University, Bangui, CAF; 3 Business and Management, University of Cumbria, Carlisle, GBR; 4 Medicine, Ohio University Heritage College of Osteopathic Medicine, Athens, USA; 5 Medicine, Yale New Haven Health, New Haven, USA; 6 Medicine, Bridgeport Hospital, Yale New Haven Health, Bridgeport, USA; 7 Internal Medicine, Columbia University, Harlem Hospital Center, New York, USA; 8 Internal Medicine, Bridgeport Hospital, Yale New Haven Health, Bridgeport, USA; 9 Internal Medicine, Baton Rouge General Medical Center, Baton Rouge, USA; 10 Medicine, Icahn School of Medicine at Mount Sinai, New York, USA; 11 Medicine, Richmond University Medical Center, Staten Island, USA; 12 Cardiology, The University of Texas Medical Branch, Galveston, USA; 13 Internal Medicine/Cardiology, Richmond University Medical Center, Staten Island, USA; 14 Biological Science, University of Maryland, Baltimore, Baltimore, USA; 15 Rehabilitation Medicine, Montefiore Medical Center, The Bronx, USA; 16 Digestive Diseases, Yale University School of Medicine, New Haven, USA

**Keywords:** acute myocardial infarction, cannabis use disorder (cud), cardiovascular outcomes, cirrhosis, in-hospital mortality, metabolic dysfunction-associated steatohepatitis (mash)

## Abstract

Introduction: Cannabis use disorder (CUD) is encountered among hospitalized adults with metabolic dysfunction-associated steatohepatitis (MASH) cirrhosis. Both conditions are independently associated with adverse cardiometabolic profiles, raising concern for potential cardiovascular complications when they coexist. This study evaluated the association between CUD and acute myocardial infarction (MI) among hospitalized adults with MASH cirrhosis.

Methods: We performed a retrospective cross-sectional analysis of the National Inpatient Sample from 2016 to 2020, including adults aged ≥18 years hospitalized with MASH cirrhosis. CUD was identified using the International Classification of Diseases, Tenth Revision, Clinical Modification (ICD-10-CM) codes. Multivariable logistic and Poisson regression models were used to examine associations between CUD and acute MI, in-hospital mortality, major adverse cardiovascular events, hepatic decompensation, length of stay, and hospitalization costs, adjusting for demographics, comorbidities, and hospital characteristics.

Results: Among 57,754 hospitalizations for MASH cirrhosis, 539 involved patients with CUD. CUD was associated with a significantly higher likelihood of acute MI (adjusted odds ratio 2.18; 95% CI 1.30-3.63). No significant associations were observed between CUD and in-hospital mortality or overall major adverse cardiovascular events. CUD was associated with lower odds of hepatic decompensation. Hospitalizations involving CUD were associated with lower adjusted costs, while length of stay did not differ significantly between groups.

Conclusion: Among hospitalized adults with MASH cirrhosis, CUD was associated with a higher risk of acute MI but not with increased in-hospital mortality or overall major adverse cardiovascular events. These findings underscore the importance of cardiovascular risk assessment and CUD identification in this population. As this was an observational cross-sectional study, the findings represent associations and do not establish causality.

## Introduction

During 2016-2020, the period covered by our study, cannabis use and legalization extended rapidly across the United States, with many states agreeing on recreational and medical use. By 2020, 33 states and the District of Columbia had legalized medical cannabis, and 11 had legalized recreational use. These changes occurred simultaneously with increasing national cannabis use, highlighting the need to understand its cardiovascular effects in high-risk populations, such as patients with metabolic dysfunction-associated steatohepatitis (MASH) cirrhosis. Our dataset captures this transitional period preceding wider legalization after 2020 [1]. Cannabis use has been associated with adverse cardiovascular outcomes, including myocardial infarction (MI), arrhythmias, heart failure, stroke, and early-onset cardiovascular disease [2-5]. This association may be partly driven by social determinants of health, as cannabis use is more common in socioeconomically disadvantaged populations with a higher baseline cardiovascular risk. The observed effect may therefore reflect an additive influence of cannabis-related triggers and underlying social and environmental risk factors [6]. Acute MI, in particular, is a leading cause of mortality and morbidity and represents the primary cardiovascular outcome of interest in this study [4]. Endocannabinoid receptors are widely presented throughout the cardiovascular system. Tetrahydrocannabinol (THC), the active component of cannabis, modulates CB1R, resulting in pro-apoptotic and pro-atherogenic effects through decreased cholesterol clearance, vascular endothelial dysfunction, increased pro-inflammatory cytokine expression, and reactive oxygen species synthesis [2,3]. Evidence suggests that the adverse cardiovascular effect of cannabis has been demonstrated to be independent and, in some studies, comparable in magnitude to those of tobacco use [2,4,7].

MASH, formerly known as nonalcoholic steatohepatitis (NASH), and cirrhosis are part of the clinical spectrum of metabolic dysfunction-associated steatotic liver disease (MASLD) [8]. MASLD remains the leading cause of chronic liver disease, as well as liver-related morbidity and mortality globally [9]. Cardiovascular disease risk is significantly increased in patients with MASH and MASH cirrhosis compared with other etiologies of cirrhosis [4,10]. This elevated risk stems from a shared bidirectional pathophysiology of systemic inflammation and endothelial dysfunction, where hepatic metabolic stress simultaneously drives a pro-atherogenic environment that predisposes patients to serious cardiovascular diseases like coronary artery disease, atrial fibrillation, heart failure, etc. [11]. The global prevalence of MASLD has risen from 25.26% in 1990-2006 to 30.8% in 2016-2019, with an estimated global MASH prevalence of 5.27% in 2023, expected to increase alongside obesity and diabetes mellitus [8]. Human and animal models have demonstrated increased CB1R expression in hepatocytes and hepatic myofibroblasts in MASH and cirrhosis, suggesting that THC-mediated CB1R activation may contribute to an increased cardiovascular risk in patients with MASH cirrhosis through steatogenic, inflammatory, and fibrogenic pathways [3,12,13].

Despite the presence of studies documenting the individual impacts of cannabis use and MASLD/MASH cirrhosis on cardiovascular outcomes, studies examining the cardiovascular impact of cannabis use disorder (CUD) specifically among hospitalized patients with MASH cirrhosis are lacking. Hospitalized individuals with MASH cirrhosis represent a high-risk subgroup characterized by a greater cardiometabolic burden and liver disease compared to the community population. Therefore, this study aims to investigate whether CUD is associated with an increased risk of acute MI in hospitalized US adults with MASH cirrhosis and whether this risk differs from that of those without CUD, utilizing data from the National Inpatient Sample (NIS). 

This study was previously presented as an abstract (Su1576) at Digestive Disease Week (DDW) 2024 (May 18-21, 2024).

## Materials and methods

Data source

This study used data from the NIS database for the years 2016-2020. As this is an observational, cross-sectional analysis of a national database, it allows the assessment of associations but does not permit inference of causal relationships. The NIS repository is part of the Healthcare Cost and Utilization Project (HCUP) and is provided by the Agency for Healthcare Research and Quality (AHRQ). It includes a representative sample of approximately 40 million annual hospital discharges across the United States. The NIS provides a comprehensive view of inpatient care, encompassing over 100 clinical and non-clinical variables for each hospital stay, such as primary and secondary diagnoses, patient demographics, comorbidities, total hospital charges, and various hospital characteristics. This database also includes detailed information on diagnoses and procedures, which, since 2016, has been coded using the International Classification of Diseases, Tenth Revision, Clinical Modification (ICD-10-CM) codes. We utilized deidentified patient information from the NIS database. As a result, it was deemed exempt from review by the Yale Institutional Review Board.

Study population

The study population consisted of adult patients (≥18 years) hospitalized from 2016 to 2020 with a diagnosis of MASH cirrhosis, identified using the ICD-10-CM codes. The full list of ICD-10-CM codes used to define CUD is provided in the Appendices. Patients were then categorized based on the presence or absence of a diagnosis of current CUD, identified using ICD-10-CM codes. We excluded cannabis use cases coded "in remission" from CUD cases. For all analyses, patients with CUD were compared to hospitalized MASH patients without CUD to assess differences in demographic characteristics, clinical outcomes, and comorbidities. The primary objective of this analysis was to examine the association between CUD and the risk of acute MI among hospitalized adults with MASH cirrhosis, while the secondary objective was to compare this cardiovascular risk between patients with and without CUD. According to ICD-10-CM, substances coded as cannabis use included individual use of Indian hemp, marijuana, and other varieties of cannabis and cannabinoids.

Inclusion and exclusion criteria

Inclusion criteria were adults aged 18 years and older with a primary or secondary diagnosis of MASH cirrhosis. We excluded patients with missing data on key variables such as age, sex, race/ethnicity, or primary diagnosis.

Outcome measures

The primary outcome of interest was the occurrence of MI during hospitalization. Secondary outcomes included major adverse cardiovascular events (MACEs), in-hospital mortality, hepatic decompensation, length of hospital stay, and hospitalization cost. MACEs were defined as a composite outcome including acute MI, cardiac arrest, stroke, heart failure, and atrial fibrillation. In-hospital mortality was defined using the discharge disposition recorded in the NIS (death during hospitalization vs. survival to discharge). The cost of hospitalization was calculated using the total charges provided by the NIS and modified to cost estimates using cost-to-charge ratios provided by the HCUP. All costs were then constant 2020 US dollars using the suitable cost deflators.

Covariates

Covariates included demographic variables (age, sex, race/ethnicity), smoking status, cocaine use, hospital characteristics (size, teaching status, urban/rural location), admission type (elective or non-elective), and year of admission. Comorbid conditions were identified using the Elixhauser Comorbidity Index, which includes 31 categories of comorbidities. Additional covariates included prior MI, sarcopenia, and socioeconomic factors. We did not include data on concurrent medication use as benzodiazepines or opioids, due to limitations in the NIS database regarding the reliable capture of prescribed outpatient medications. We acknowledge that some patients with CUD may have been medicinal cannabis users, potentially taking these medications for sleep or pain disorders, which could contribute to confounding.

Statistical analysis

Descriptive statistics were used to summarize the characteristics of the study population. Continuous variables were reported as medians with interquartile range and compared using the Wilcoxon log-rank test. Categorical variables were reported as counts and percentages and compared using the chi-squared test.

Multivariable logistic regression models were used to assess the association between CUD and the risk of acute MI, adjusting for potential confounders. In all analyses, patients with CUD were compared to hospitalized MASH patients without CUD to evaluate differences in outcome while adjusting for demographic, hospital-level, and clinical covariates. Multivariable logistic and Poisson regression models were used to analyze categorical and continuous secondary outcomes, respectively. Adjusted odds ratios (aORs) or incidence rate ratios (aIRRs) and 95% confidence intervals (CIs) were reported for all models. In the multivariable analyses, adjustments were made for age, gender, race, Elixhauser Comorbidity Index, prior MI, sarcopenia, income levels, and hospital characteristics. Costs were adjusted to 2020 dollars to account for inflation, using the appropriate economic deflators.

All statistical analyses accounted for the complex survey design of the NIS, including clustering and stratification. Discharge weights provided by the NIS were used to produce national estimates. Analyses were performed using the SAS software, Version 9.4 (SAS Institute Inc., Cary, North Carolina, United States) and R software, Version 4.1.0 (R Core Team, R Foundation for Statistical Computing, Vienna, Austria).

## Results

Patient characteristics and hospital data

A total of 57,754 hospitalized US adults with MASH cirrhosis were included in the analysis, of which 539 patients (0.43%) had a diagnosis of CUD. The median age of patients was 60 years, and the majority were female (58%) and non-Hispanic white (76%).

Patients with CUD were younger, with a median age of 54 years (IQR 13), compared to 66 years (IQR 14) in patients without CUD (p<0.001). The CUD group had a higher proportion of males (47.6% vs. 36.9%; p=0.003). The racial composition was similar between the groups, though a higher percentage of Hispanic patients was observed in the CUD group (17.5% vs. 13.8%; p=0.374).

Table 1 summarizes the baseline characteristics of the study population. Notably, CUD patients had a higher prevalence of hypertension (39.8% vs. 32.5%; p=0.039) and lower prevalence of cardiac arrhythmia (13.6% vs. 21.8%; p=0.008) and chronic kidney disease (20.9% vs. 37%; p<0.001). The prevalence of diabetes, obesity, prior MI, and stroke/transient ischemic attack (TIA) did not differ significantly between the groups.

**Table 1:** Characteristics of hospitalized US adults with MASH cirrhosis, with/without CUD MASH: metabolic dysfunction-associated steatohepatitis; CUD: cannabis use disorder; IQR: interquartile range; TIA: transient ischemic attack; PCI: percutaneous coronary intervention; CABG: coronary artery bypass grafting; COPD: chronic obstructive pulmonary disease; LOS: length of stay

Variable	CUD (N=539)	No CUD (N=57,215)	P-value	Test statistic
Patient characteristics				
Age, median (IQR), in years	54 (13)	66 (14)	<0.001	Wilcoxon rank-sum (Z=13.73)
Gender				
Male	257 (47.6%)	21,111 (36.9%)	0.003	Chi-square (chi^2^=8.93 (df=1))
Female	282 (52.4%)	36,104 (63.1%)		
Current smoker	27 (5%)	2,289 (4%)	0.787	Chi-square (chi^2^=0.07 (df=1))
Race/ethnicity				
White	396 (73.5%)	44,834 (78.4%)	0.374	Chi-square (chi^2^=3.12 (df=3))
Black	20 (3.7%)	1,544 (2.7%)		
Hispanic	94 (17.5%)	7,895 (13.8%)		
Other	29 (5.3%)	1,942 (5%)		
Charlson Comorbidity Index score ≥3	446 (82.7%)	50,342 (88%)	0.033	Chi-square (chi^2^=4.57 (df=1))
Comorbidities				
Diabetes	121 (22.5%)	14,592 (25.5%)	0.785	Chi-square (chi^2^=0.07 (df=1))
Hypertension	215 (39.8%)	18,609 (32.5%)	0.039	Chi-square (chi^2^=4.26 (df=1))
Hyperlipidemia	181 (33.5%)	22,643 (39.6%)	0.098	Chi-square (chi^2^=2.73 (df=1))
Obesity	198 (36.7%)	19,554 (34.2%)	0.518	Chi-square (chi^2^=0.42 (df=1))
Cardiac arrhythmia	73 (13.6%)	12,476 (21.8%)	0.008	Chi-square (chi^2^=7.11 (df=1))
Valvular disease	1 (0.1%)	1,088 (1.9%)	0.093	Chi-square (chi^2^=2.82 (df=1))
Peripheral vascular disease	17 (3.1%)	2,289 (4%)	0.665	Chi-square (chi^2^=0.19 (df=1))
Coagulopathy	217 (40.3%)	24,100 (42.1%)	0.672	Chi-square (chi^2^=0.18 (df=1))
Carotid artery disease	79 (14.7%)	12,985 (22.7%)	0.01	Chi-square (chi^2^=6.59 (df=1))
Prior myocardial infarction	31 (5.8%)	3,373 (5.9%)	0.924	Chi-square (chi^2^=0.01 (df=1))
Prior TIA/stroke	17 (3.1%)	3,261 (5.7%)	0.178	Chi-square (chi^2^=1.81 (df=1))
Prior PCI	1 (0.1%)	343 (0.6%)	0.56	Chi-square (chi^2^=0.34 (df=1))
Prior CABG	9 (1.6%)	2,861 (5%)	0.043	Chi-square (chi^2^=4.08 (df=1))
Hypothyroidism	113 (20.9%)	13,971 (24.4%)	0.312	Chi-square (chi^2^=1.02 (df=1))
COPD	161 (29.8%)	12,930 (22.6%)	0.022	Chi-square (chi^2^=5.25 (df=1))
Chronic kidney disease	113 (20.9%)	21,174 (37%)	<0.001	Chi-square (chi^2^=20.33 (df=1))
Lymphoma	3 (0.5%)	458 (0.8%)	0.687	Chi-square (chi^2^=0.16 (df=1))
Solid tumor without metastases	23 (4.2%)	4,174 (7.3%)	0.128	Chi-square (chi^2^=2.32 (df=1))
Metastatic cancer	20 (3.7%)	974 (1.7%)	0.08	Chi-square (chi^2^=3.07 (df=1))
Type of admission				
Elective admission	23 (4.2%)	4,464 (7.8%)	0.088	Chi-square (chi^2^=2.90 (df=1))
Weekend admission	119 (22%)	12,762 (22.3%)	0.923	Chi-square (chi^2^=0.01 (df=1))
Year of admission				
2016	37 (6.8%)	8,471 (14.8%)	<0.001	Chi-square (chi^2^=24.61 (df=4))
2017	82 (15.2%)	9,830 (17.2%)		
2018	110 (20.4%)	11,831 (20.7%)		
2019	113 (20.9%)	13,244 (24.2%)		
2020	197 (36.7%)	13,244 (23.8%)		
LOS, median (IQR), days	4 (5)	4 (5)	0.575	Wilcoxon rank-sum (Z=-0.56)
Overall cost, median (IQR), USD, $	37,803 (48,430)	41,849 (50,173)	0.149	Wilcoxon rank-sum (Z=1.44)
Discharge disposition				
Routine discharge or home self-care	454 (84.3%)	44,557 (77.9%)	0.018	Chi-square (chi^2^=8.03 (df=2))
Short-term hospital	25 (4.7%)	1,945 (3.4%)		
Skilled nursing facility	60 (11%)	9,513 (16.7%)		
Primary payer source				
Private insurance	221 (41%)	36,885 (64.5%)	<0.001	Chi-square (chi^2^=109.86 (df=5))
Medicaid	170 (31.6%)	5,775 (10.1%)		
Medicare	114 (21.1%)	12,231 (21.4%)		
Other payment source	25 (4.7%)	1,088 (1.9%)		
Self-pay	1 (0.1%)	114 (0.2%)		
No charge	8 (1.5%)	1,088 (1.9%)		
Median household income, $				
<38,999	194 (36%)	17,786 (31.1%)	0.253	Chi-square (chi^2^=4.08 (df=3))
39,000-47,999	168 (31.2%)	16,649 (29.1%)		
48,000-62,999	106 (19.6%)	13,976 (24.4%)		
>63,000	71 (13.2%)	8,804 (15.4%)		
Location/teaching status				
Rural	14 (2.6%)	4,177 (7.3%)	0.024	Chi-square (chi^2^=7.46 (df=2))
Urban non-teaching	82 (15.2%)	9,957 (17.4%)		
Urban teaching	443 (82.2%)	43,081 (75.3%)		
Hospital size				
Small	71 (13.1%)	9,832 (17.2%)	0.331	Chi-square (chi^2^=2.21 (df=2))
Medium	141 (26.2%)	14,577 (25.5%)		
Large	327 (60.7%)	32,806 (57.3%)		

**Figure 1:**
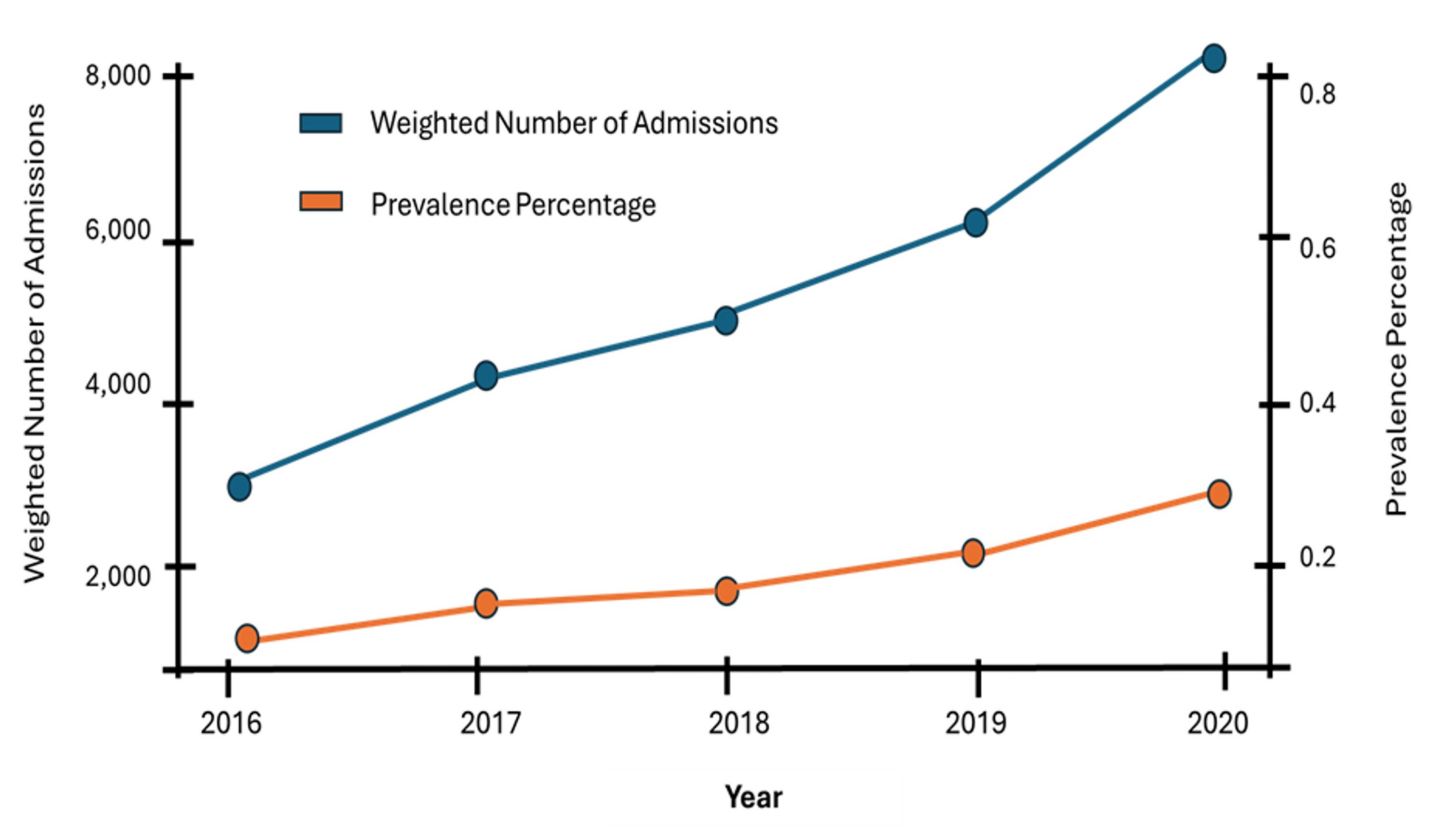
Weighted admissions of MASH cirrhosis patients with CUD by year MASH: metabolic dysfunction-associated steatohepatitis; CUD: cannabis use disorder

Figure 1 illustrates the weighted annual hospital admissions for MASH cirrhosis patients with CUD from 2016 to 2020. The trend over the five-year period reveals a consistent year-to-year increase in the number and percentage of admissions with MASH cirrhosis with CUD. There were an estimated 3,200 national admissions of CUD in patients with MASH cirrhosis (0.12% of all MASH cirrhosis admissions) in 2016. This number increased to an estimated 8,100 national admissions (0.3% of all MASH cirrhosis admissions) in 2020.

Clinical outcomes

**Figure 2:**
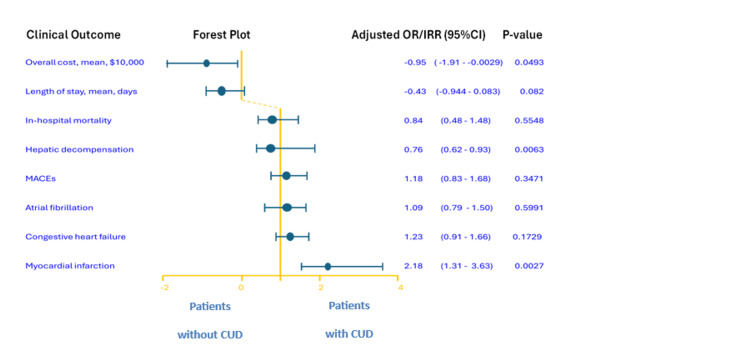
Forest plot of adjusted odds ratios for clinical outcomes in hospitalized US adults with MASH cirrhosis and CUD MASH: metabolic dysfunction-associated steatohepatitis; CUD: cannabis use disorder; MACEs: major adverse cardiovascular events

Table 2, Table 3, and Figure 2 show the clinical outcomes of hospitalized patients with MASH cirrhosis with and without CUD. In the multivariable logistic regression model, CUD was significantly associated with an increased risk of acute MI. The aOR for acute MI in patients with CUD was 2.18 (95% CI: 1.30-3.63), indicating a 2.2-fold increased risk compared to those without CUD (p<0.001).

**Table 2:** Association between CUD and cardiovascular outcomes in MASH cirrhosis patients: binary outcomes MASH: metabolic dysfunction-associated steatohepatitis; CUD: cannabis use disorder; ACS: acute coronary syndrome; Afib: atrial fibrillation; MACE: major adverse cardiovascular event

Outcome	Type	Odds ratio	95% lower CI	95% upper CI	P-value
Cardiac arrest	Unadjusted	0.857	0.274	2.679	0.7916
Cardiac arrest	Adjusted	0.726	0.228	2.315	0.5885
ACS	Unadjusted	1.434	0.786	2.616	0.2393
ACS	Adjusted	2.309	1.230	4.335	0.0092
Acute MI	Unadjusted	1.399	0.861	2.274	0.1750
Acute MI	Adjusted	2.180	1.310	3.627	0.0027
Heart failure	Unadjusted	0.567	0.451	0.714	<0.0001
Heart failure	Adjusted	1.230	0.913	1.658	0.1729
Decompensated liver	Unadjusted	0.442	0.371	0.527	<0.0001
Decompensated liver	Adjusted	0.759	0.622	0.925	0.0063
Afib	Unadjusted	0.502	0.372	0.677	<0.0001
Afib	Adjusted	1.090	0.791	1.502	0.5991
MACE	Unadjusted	0.571	0.466	0.700	<0.0001
MACE	Adjusted	1.182	0.834	1.676	0.3471
Died	Unadjusted	0.605	0.348	1.050	0.0801
Died	Adjusted	0.844	0.481	1.482	0.5548

**Table 3:** Association between CUD and cardiovascular outcomes in MASH cirrhosis patients: continuous outcomes MASH: metabolic dysfunction-associated steatohepatitis; CUD: cannabis use disorder

Outcome	Type	Incidence rate ratio	95% lower CI	95% upper CI	P-value
Inflation-adjusted charges, ($)	Unadjusted	-6785.905	-16498.003	2926.19	0.1709
Inflation-adjusted charges, ($)	Adjusted	-9553.6477	-19077.8324	-29.4630	0.0493
Length of stay (days)	Unadjusted	-0.446	-0.973	0.0802	0.0967
Length of stay (days)	Adjusted	-0.430	-0.944	0.0832	0.0828

There was no significant difference in in-hospital mortality between patients with and without CUD (aOR=0.77; 95% CI: 0.31-1.90; p=0.567). Similarly, no significant association was found between CUD and the overall MACEs, which included acute MI, cardiac arrest, stroke, heart failure, and atrial fibrillation.

Length of stay and hospitalization costs

The analysis of continuous outcomes showed lower hospitalization costs for patients with CUD by $9,553.65 (95% CI: -19077.83 to -29.46; p<0.0493) when adjusted. The median length of hospital stay was similar between patients with and without CUD.

## Discussion

We aimed to investigate whether CUD is associated with an increased risk of acute MI among hospitalized adults with MASH cirrhosis and whether this risk differs from that of those without CUD. CUD was associated with a twofold increased risk of acute MI after controlling for cardiometabolic comorbidities and hospital characteristics. Notably, the non-CUD group had a higher prevalence of MI-related comorbidities, including coagulopathy and coronary artery disease. These findings suggest that CUD may function as an independent cardiovascular risk factor in patients with MASH cirrhosis. To our knowledge, no prior study has specifically examined this population, highlighting a clear gap in the literature.

Our results extend and align with prior research linking cannabis use to cardiovascular events in the general population. It is important to note that most previous studies focused on general cannabis use rather than clinically diagnosed CUD. For example, Jeffers et al. found an increased risk of stroke and composite outcomes (coronary heart disease, MI, stroke) in daily adult cannabis smokers [2]. Similarly, a cross-sectional study involving 33,173 young adults utilizing data from the American Behavioral Risk Factor Surveillance System found an increased risk of MI (aOR 2.07; 95% CI 1.12-3.82) in recent cannabis users, with smoking and frequent use (>4 times/month) associated with higher odds [5]. Chami and Kim observed a 72% increased MI risk among patients with cannabis abuse in a large multicenter cohort (aOR 1.72; 95% CI 1.36-2.18) [14]. In comparison, the association observed in our study (aOR 2.18; 95% CI 1.30-3.63) was of greater magnitude, suggesting that the pro-inflammatory and metabolic environment of MASH may heighten vascular vulnerability to cannabis-related cardiovascular injury through mechanisms such as autonomic stimulation, platelet activation, endothelial dysfunction, and coronary vasospasm.

Interestingly, unlike some broader population studies reporting increased risk of composite cardiovascular events, we did not observe a significant association with overall MACEs. This secondary finding may reflect competing risks in advanced cirrhotic patients. The observed association between CUD and MI, but not with overall MACEs, likely reflects that THC's cardiovascular effects act as acute triggers for ischemic events, whereas other MACE components are influenced by chronic cardiac remodeling, not captured during hospitalization. This difference highlights that acute MI may be the most sensitive manifestation of cannabis-related cardiovascular stress in the setting of MASH cirrhosis.

MASH cirrhosis and CUD have each been associated with increased cardiovascular risk [2,4,5,15]. MASH is characterized by metabolic dysfunction and chronic systemic inflammation, both of which accelerate atherosclerosis and increase the chance of acute MI [16]. Individuals with CUD may have an increased risk of acute MI through physiological mechanisms. Endocannabinoid dysregulation may impair myocardial perfusion, particularly in obese patients [17]. Combined with systemic inflammation and metabolic comorbidities in MASH, these effects likely amplify cardiovascular risk [2,3,10,15].

Our study has some strengths and limitations. The primary strength of our study lies in its use of the NIS, a large and nationally representative database, which offers a robust platform for analyzing hospitalizations of patients with MASH cirrhosis across the United States. This allows for a comprehensive evaluation of epidemiological patterns, outcomes, and complications associated with CUD among patients with MASH cirrhosis. The extensive data, covering patient demographics, hospital characteristics, and baseline comorbidities, enable a thorough understanding of clinical outcomes and implications. Despite these strengths, there are notable limitations to consider. First, this was an observational, cross-sectional study using administrative data; therefore, causality cannot be inferred from the observed associations. Rigorous prospective studies are required to validate these findings and confirm causality before they can influence clinical practice. The retrospective nature of the NIS database means it lacks specific data on the severity and progression timeline of MASH cirrhosis and cannabis use, as well as detailed information on patient medication regimens. Notably, information on medications such as opioids and benzodiazepines is not captured, which may be relevant since some patients with CUD may use medicinal cannabis for pain or sleep. This represents a potential unmeasured confounder. The reliance on ICD codes to identify CUD and other conditions introduces the potential for misclassification and bias inherent in any retrospective analysis. Furthermore, the study's focus on hospitalizations rather than individual patient outcomes may inadvertently include multiple admissions for the same patient, potentially skewing incidence rates and outcomes. The use of administrative data, a characteristic of the NIS, also carries the risk of coding inaccuracies, which could affect our findings. Lastly, the NIS database, while extensive, is limited to inpatient data, excluding post-discharge outcomes that are crucial for a comprehensive understanding of patient trajectories following hospitalization for MASH cirrhosis with CUD. Our findings highlight the need for cardiovascular risk assessment and cannabis use screening in patients with MASH cirrhosis. Clinicians should counsel patients on the potential cardiac risks of cannabis, especially frequent or smoked use. These results also call for further research on the mechanisms linking cannabis use, liver disease, and cardiovascular injury. Additionally, this is the first study to specifically assess the cardiovascular impact of CUD in this population, emphasizing the existing literature gap and the novelty of our findings.

The main strength of this study is the use of a large, nationally representative inpatient database, which enhances generalizability and allows for the evaluation of a diverse population of patients with MASH cirrhosis. Additionally, this is the first study to specifically assess the cardiovascular impact of CUD in this population. However, important limitations include the retrospective design and reliance on ICD-10 coding, which may introduce misclassification. It is important to note that the cannabis landscape has changed significantly since the study period (2016-2020) and will continue to evolve, which may influence the prevalence of CUD and its associated cardiovascular risks in hospitalized patients with MASH cirrhosis. The NIS database does not provide information on cannabis dose, route, frequency, or duration of use nor on liver disease severity measures such as the Model for End-Stage Liver Disease (MELD) or Child-Pugh scores. We also could not assess long-term or post-discharge outcomes. Therefore, while our findings show an association, they do not establish causality, and prospective studies are needed. These findings have important implications for frontline researchers and clinicians. They highlight the need for integrated cardiovascular risk assessment and cannabis use screening in patients with MASH cirrhosis and emphasize the novelty of our study as the first to specifically examine CUD in this high-risk population.

## Conclusions

This study is the first, to our knowledge, to specifically evaluate the cardiovascular impact of CUD in hospitalized adults with MASH cirrhosis using a large, nationally representative US inpatient database. Our study indicates that CUD is associated with an increased risk of MI in patients with MASH cirrhosis. The observed increase in risk was higher than reported in previous studies documenting an association between cannabis use and cardiovascular disease risks in the general population. These findings emphasize the importance of heightened surveillance for CUD in patients with MASH cirrhosis and suggest the need for cardiovascular monitoring and patient education in this population.

## Appendices

**Table 4:** The full list of ICD-10-CM codes used to define CUD ICD-10-CM: International Classification of Diseases, Tenth Revision, Clinical Modification; CUD: cannabis use disorder

ICD-10-CM code	Description
F12.10	Cannabis abuse, uncomplicated
F12.120	Cannabis abuse with intoxication, uncomplicated
F12.121	Cannabis abuse with intoxication delirium
F12.122	Cannabis abuse with intoxication with perceptual disturbance
F12.129	Cannabis abuse with other cannabis-induced disorder
F12.130	Cannabis abuse with withdrawal
F12.138	Cannabis abuse with other cannabis-induced disorder
F12.139	Cannabis abuse with unspecified cannabis-induced disorder
F12.180	Cannabis abuse with cannabis-induced anxiety disorder
F12.188	Cannabis abuse with other cannabis-induced disorder
F12.19	Cannabis abuse with unspecified cannabis-induced anxiety disorder
F12.20	Cannabis dependence, uncomplicated
F12.21	Cannabis dependence, in remission
F12.22	Cannabis dependence with intoxication
F12.23	Cannabis dependence with withdrawal
F12.24	Cannabis dependence with cannabis-induced psychotic disorder
F12.25	Cannabis dependence with cannabis-induced anxiety disorder
F12.26	Cannabis dependence with other cannabis-induced disorder
F12.29	Cannabis dependence with unspecified cannabis-induced disorder
